# Developing phytocompound-based new drugs against multi-drug-resistant *Staphylococcus aureus*

**DOI:** 10.1098/rsos.231475

**Published:** 2024-07-24

**Authors:** Md. Nazmussakib Shuvo, Sajal Kumar Halder, Nuhu Alam, Mahbubul Kabir Himel, Aparna Shil

**Affiliations:** ^1^ Department of Botany, Jahangirnagar University, Savar, Dhaka 1342, Bangladesh; ^2^ Padma Bioresearch, Dhaka 1342, Bangladesh

**Keywords:** phytocompounds, *Staphylococcus aureus*, pharmacokinetics, drug-likeness, therapeutics

## Abstract

*Staphylococcus aureus*, a prevalent component of the human microbiota, is associated with skin infections to life-threatening diseases, presenting challenges in treatment options and necessitating the development of effective treatments. This study integrated computational and *in vitro* approaches to identify promising phytocompounds with therapeutic potential. Staphopain B emerged as a target protein for its role in immune evasion, exhibiting stability during molecular dynamic simulation (MDS) with a root mean square deviation value of 2.376 Å. Screening 115 phytocompounds with antibacterial properties from the PubChem database identified 12 with drug-like properties, nine of which showed superior binding affinity to Staphopain B compared to a commercial antibiotic, doxycycline (−7.8 kcal mol^−1^). Notably, epoxyazadiradione and nimbolide displayed higher estimated free energy of binding scores (−7.91 and −7.93 kcal mol^−1^, respectively), indicating strong protein–ligand interactions. The root mean square fluctuation values for epoxyazadiradione and nimbolide were 1.097 and 1.034 Å, respectively, which was confirmed through MDS. Crude ethanolic extracts (100% and 70%) of neem (*Azadirachta indica*) leaves demonstrated narrow inhibition against the bacteria in comparison to doxycycline in the disc-diffusion assay. This study underscores the potential of phytocompounds as therapeutic agents against *S. aureus*; however, further *in vitro* experiments and testing of the phytocompounds *in vivo* are required.

## 1. Introduction

*Staphylococcus aureus* is a Gram-positive, cocci-shaped bacterium that is typically a resident opportunistic pathogen of animal and human skin and mucosa [1]. These species inhabit healthy people’s skin, and their ratio depends on the condition of the skin [2]. Unfortunately, the primary source of habitat for *S. aureus* is hospitals. It can be transmitted from individual to individual by various commodities such as ward dust, blankets, clothes and numerous lesions [3]. It can cause mild skin infections to life-threatening ones like toxic shock syndrome, endocarditis, arthritis, osteomyelitis and sepsis [4,5]. Moreover, it shows resistance to different types of antibiotics, for example, methicillin, aminoglycosides, tetracyclines, trimethoprim-sulfamethoxazole, erythromycin, rifampicin, vancomycin and macrolides [6–9]. This antibiotic resistance is more fatal; infections caused by antibiotic-resistant *S. aureus* are 64% deadlier than those caused by drug-sensitive strains of the bacteria [10]. Other than acquiring antibiotic resistance, it pursues a variety of tactics to get away from the defence system of the body. Staphopain B, a cysteine proteinase, is one of the agents that help bacteria escape from phagocytes.

Among the two papain-like proteases, Staphopain A and B, derived from human strains of *S. aureus*, Staphopain B contributes to bacterial virulence while Staphopain A possibly plays a house-keeping function [11]. CD11b on phagocytes found in the peripheral circulation can be cleaved by Staphopain B, which causes the fast development of characteristics associated with the death of those cells. Staphopain B also works as an antiphagocytic agent by inhibiting the chemotactic activity of monocytes and neutrophils. Furthermore, an antiphagocytic signal, CD31, which remains on the surface of neutrophils and represents the ‘do not-eat-me’ signal, is cleaved by the protein [12]. As a consequence of this, treating the protein as a target and inhibiting its function have the potential to assist with the long-term control and elimination of staphylococcal infections [13]. Previously, lapatinib and efavirenz have emerged as promising agents in combating the biofilm formation and virulence of *S. aureus* by targeting the gene *staphopain B* [14,15]. In addition, the cysteine proteases, collectively known as staphopains, play a central role in the intricate processes of biofilm dynamics, with Staphopain B being a key contributor. Squamous Cell Carcinoma Antigen 1, an epithelial-derived serpin, efficiently inhibits staphopains, underscoring a potential avenue for therapeutic intervention [16]. Furthermore, Staphopain B is accountable for the connective tissue degradation, kinin systems and clotting, which allows it to come into direct touch with the immune cells of the host. In light of the fact that these proteases play an important enzymatic role and have the potential to play a role in the destruction of biofilms, the dibenzyl(benzo[*d*]thiazol-2-yl(hydroxy)methyl) phosphonate has demonstrated anti-*S. aureus* and anti-biofilm characteristics by increasing the production of proteases [17]. So, it has become increasingly important to continue attempts to produce an inhibitory molecule derived from naturally occurring plant elements that target the protein.

Plant elements, or phytocompounds, in particular, have been used by humans as the first line of defence against diseases. Historical records and cultural practices have revealed a long legacy of using medicinal plants and other natural remedies as the foundation for primary healthcare [18]. In approximately 2600 BC, Mesopotamia was the place where the first extracts were used for therapeutic purposes [19]. The secondary metabolites that remain in plant extracts aid in human defence against numerous diseases and provide the body with protection since historical times [20]. For example, the extract of the lichen *Parmelia omphalodes* was used in the treatment of burn patients and cuts in Europe [21]. Particularly with regard to breast cancer, the juice extracted from the red alga *Porphyra umbilicalis* has been identified for its ability to inhibit the growth of cancerous cells [22]. Furthermore, the bark, leaf and oil extracts of the neem tree have all been used medicinally for a variety of purposes, including the treatment of constipation, respiratory diseases, intestinal helminthiasis and leprosy; also, neem oil has been used as a general health booster [23]. So, medicinal plant extracts have been used for therapeutic purposes since ancient times.

Recently, competent therapeutic phytocompounds have been developed using computer-aided systems within a short time. To produce medicines and other pharmacologically active compounds, researchers are now turning to computer-assisted drug development (CADD), which involves the use of computational methods for drug discovery, design and evaluation [24]. An eye-catching difference has been observed in compound screening by CADD over time. For instance, prediction of the structure of the target and making their model, indication of the active site, comprising protein–ligand complexes, assessing a massive number of substances in a dataset by evaluating their drug-likeness properties and observing the binding stability of protein–ligand complexes are promptly done by CADD [25]. The interaction between protein and ligand can be established by molecular docking and validated by binding affinity score or estimated ΔG score. The ΔG value offers valuable information regarding the thermodynamic favourability of the binding interaction [26].

The purpose of this study is to identify phytocompounds from native plants that are effective against Staphopain B protein, to evaluate their binding affinity with the protein using molecular docking and stability using molecular dynamics (MD) simulation, and to assess their absorption, distribution, metabolism, excretion and toxicity using a computational method. Additionally, the efficacy of the selected phytocompounds in inhibiting *S. aureus* was also evaluated.

## 2. Material and methods

**Figure 1.**
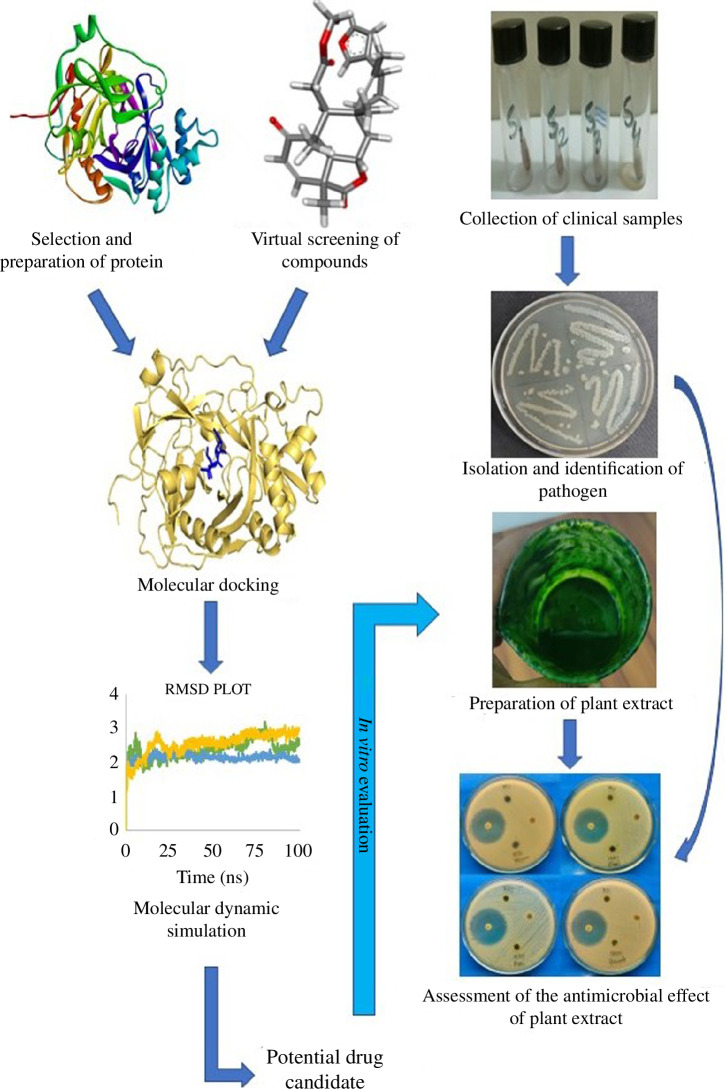
The complete overview of the work. Both computational and *in vitro* approaches were used to identify potential drug candidates against *S. aureus*.

The research was conducted through *in silico* and *in vitro* approaches. The complete design is introduced in figure 1.

### 2.1. *In silico* approach

#### 2.1.1. Target protein selection

Staphopain B was selected as a target protein from the Protein Data Bank website (https://www.rcsb.org/) because of its morphology, including the number of amino acids, and its importance in the survivability of the pathogen [12,27]. The FASTA sequence of the protein was retrieved from the UniProt website (https://www.uniprot.org/). As the protein has a template (1X9Y), which has 100% identity and 0.92 GMQE value (https://swissmodel.expasy.org/), homology modelling was done by the Modeller tool [28]. The stability and flexibility of the protein were also determined by MD simulation [29].

#### 2.1.2. Phytocompound selection

A total of 115 phytocompounds were retrieved from Dr. Duke’s phytochemical and ethnobotanical databases (https://phytochem.nal.usda.gov/phytochem/search/list) and the natural product activity and species source database (https://bidd.group/NPASS/), which are the components of 14 indigenous medicinal plants of Bangladesh. These plants have several antimicrobial activities against *S. aureus* [30,31].

#### 2.1.3. Determination of drug-likeness properties

Drug-likeness refers to the structural and physicochemical properties of a drug-like molecule [32]. The analysis of pharmacokinetic properties, physiochemical descriptors and drug-likeness properties of those selected phytocompounds was done by the SWISS ADME (http://www.swissadme.ch/) Web server, which is free to access [33,34]. The Canonical SMILES of phytocompounds were retrieved from the PubChem database (https://pubchem.ncbi.nlm.nih.gov/) and used in the SWISS ADME server. This server applies five rules, namely those of Lipinski [35], Muegge [36], Veber [37], Ghose [38] and Egan [39], to assess the findings. The 115 phytocompounds that showed antimicrobial properties previously were submitted for initial screening.

#### 2.1.4. Determination of ADMET properties

The analysis of various physiological properties such as absorption, distribution, metabolism, excretion and toxicity of ligands is called ADMET analysis. A Web server named pkCSM (https://biosig.lab.uq.edu.au/pkcsm/prediction) is an effective tool for forecasting these characteristics of small compounds [40]. Several parameters, including human intestinal absorption and Caco−2 permeability, blood–brain barrier, bioavailability, excretion, CYP2D6 substrate, CYP1A2 inhibitor, CYP2C19 inhibitor, CYP2C3A4 inhibitor, P gp-I inhibitor, P gp-II inhibitor and drug–drug interactions were determined by the server. To assess toxicity, including hepatotoxicity, hERG inhibitors, AMES toxicity and maximum tolerated dosage, a variety of computational techniques were applied.

#### 2.1.5. Molecular docking through Autodock-vina

The chemical structures of ligands, including phytocompounds and antibiotics that are prevalently used for treatment purposes, were obtained from the PubChem database. These ligands were converted to the Autodock-vina-supported pdbqt format by the molecular graphics laboratory tool and then minimized by the UCSF Chimera [41,42]. Following that, the protein was placed on the Autodock-Vina to add polar hydrogen bonds and transform it from pdb to pdbqt format [43]. Following the process of minimizing the protein model, the CASTp 3.0 (https://sts.bioe.uic.edu/castp/) server was used to locate the active site of the protein [44]. Multiple binding sites were predicted with amino acids (sequence nos. 54, 66, 80, 101, 102, 103, 105, 130, 142,144, 323, 325, 327, 328, 332, 341, 342, 343 and 344). The information from the binding pocket was used to determine the location of the centre of the grid box for Staphopain B. This centre was placed at the coordinates *X* = 15.595, *Y* = 13.350 and *Z* = 32.637, and it covers most of the binding sites of the protein. The dimensions of the grid box were *X* = 90 Å, *Y* = 90 Å and *Z* = 90 Å, with a spacing of 0.375 between each grid point. The program was executed from the command prompt, and the results provided nine different poses. The position with the greatest score for negative affinity was chosen. As a result, the output file, including all the data pertaining to the protein’s functionality and stability, was stored [45].

#### 2.1.6. Molecular docking through Swissdock server

The Swissdock service has a simple and easy-to-use graphical user interface for analysing protein–ligand docking [46]. Open Babel software was used to convert the ligands to MOL2 format for the Swissdock server [47]. In order to accurately portray the binding interaction, the server relies on full-fitness and an estimated value of ΔG.

#### 2.1.7. Molecular dynamics simulation

To learn more about the binding stability of Staphopain B_Nimbolide, Staphopain B_Epoxyazadiradione and Staphopain B_Doxycycline complexes, MD simulation was performed in Desmond on the Linux operating system [48]. The structures of the protein–ligand complexes were hydrated using the system development tool on the cubic 3-point transferable interaction potential [49]. The generated model was then normalized to the physiological salt concentration of 0.15 M by adding Na^+^ and Cl^−^ charged ions [44]. In order to achieve maximum efficiency in terms of energy consumption, the integrated OPLS3e force field was used. The MD simulation was carried out using the isothermal isobaric composition (NPT) method at a temperature of 310 K and a pressure of 1.013 bar [48]. During the 300 ns period, the capturing interval lasted for 100 ps. In the meantime, a thousand frames were saved in the memory of the trajectory. The efficacy of the phytocompounds was tested in *in vitro* condition.

### 2.2. *In vitro* approach

#### 2.2.1. Collection and identification of *Staphylococcus aureus*

*Staphylococcus aureus* strain was isolated from clinical samples collected from patients visiting Jahangirnagar University Medical Centre. Pus from patients with skin infections was collected using a sterile syringe and cotton buds. Samples were transferred into sterile containers. The purpose of the sampling was mentioned to the patients and sampling was done with verbal permission. To verify, samples were spread on differential and selective media such as mannitol salt agar (MSA) and Gram staining and routine biochemical tests, including starch hydrolysis and catalase tests, were performed. Afterwards, an antibiotic sensitivity test of the isolated strain was done to determine a reference antibiotic.

#### 2.2.2. Antibiotic sensitivity test of the strain

The sensitivity of the organism to doxycycline, aztreonam, linezolid, clindamycin, vancomycin, oxacillin and co-trimoxazole was tested using the disc agar diffusion method, as described by Acar in 1980 and Bauer *et al*. in 1966 [50,51]. Briefly, a single colony collected from an overnight incubated culture on TSA medium was inoculated into fresh liquid media and incubated for 3–4 h to get a growth rate of about 10⁶ CFU ml^−1^. On Mueller–Hinton agar, a lawn was made using this culture. The antibiotic susceptibility pattern against seven antibiotics (purchased from HiMedia and BioMaxima SA) was observed using 6 mm filter paper discs. The amount of each antibiotic on each disc was given in micrograms (µg): 30 units of doxycycline, 30 units of aztreonam, 30 units of linezolid, 2 units of clindamycin, 30 units of vancomycin, 1 unit of oxacillin and 25 units of co-trimoxazole. For each treatment, the diameter of the zone that was created owing to bacterial inhibition (including the disc) was measured and compared to the guidelines of Clinical and Laboratory Standards Institute (CLSI) standards [52]. This gave a picture of how drugs worked compared to how resistant they were to antibiotics and helped choose a control drug [50].

#### 2.2.3. Preparation of the plant extracts

Plant extracts were prepared from neem (*Azadirachta indica*), which has antibacterial effects described in many studies. Plant leaves were collected and washed with clean water. Leaves were air-dried on a clean sheet for one week at room temperature and ground to make powder. A Soxhlet device was used for retrieving extracts from the powder. Ethanol was used to extract dried powdered leaves completely in a Soxhlet device over the course of around 12 h [48]. Afterwards, the solvent, ethanol, was evaporated with the help of a rotary evaporator, under reduced pressure at 45°C, to obtain the crude plant extract that remained in the bottom of the flask. Subsequently, to analyse the phytochemicals, the extracts were looked at under both visible and ultraviolet light using a UV spectrophotometer. The distinctive peaks in the extracts were found by performing a scan in the wavelength range of 190–1100 nm using a spectrophotometer (Analytik jena, Specord) and the peak values were recorded.

#### 2.2.4. High-performance liquid chromatography

High-performance liquid chromatography (HPLC) analysis was conducted at room temperature using a Hewlett-Packard 1100 Series HPLC system (Hewlett-Packard, CA, USA). This system consisted of a binary pump employing the mixing principle at high pressure and a UV–visible spectrophotometer detector with variable wavelength capability. The injections were made with the help of a manual injector (Rheodyne HP 7725) along with a 20 μl sample loop (Rheodyne, CA, USA). The chromatographic column used was a Lichrospher RP-18 (Supelco/Sigma-Aldrich, PA, USA) measuring 250 mm × 4.6 mm (inner diameter = 5 mm), packed with a C18 stationary phase. Data acquisition and processing were performed using HP ChemStation software for LC systems (Agilent Technologies, CA, USA). The flow rate was set at 1 ml min^−1^, with the UV detector configured to a wavelength of 217 nm [53]. A total of five HPLC injections, each lasting 36 min, were executed using a mobile phase consisting of acetonitrile with 0.1% formic acid and water for HPLC containing 0.1% of formic acid. The organic and aqueous solvents were labelled as A and B, respectively. At the start (0 min), solvent A constituted 20%, while solvent B comprised 80% of the mixture. By 9.5 min, the composition shifted to 28% A and 72% B. At the 20 min mark, the mixture was entirely composed of solvent A (100%), with no presence of solvent B. This composition remained unchanged at 25 min. By the end of 36 min, the proportions reverted to 20% A and 80% B, the same as before.

The phytocompounds were separated using a paper wick soaked in 1:1 dichloromethane and 2-propanol. the desired part was cut, dissolved in ethanol and used as a standard for the study.

#### 2.2.5. Assessment of the antibacterial effect of the plant extract

**Figure 2.**
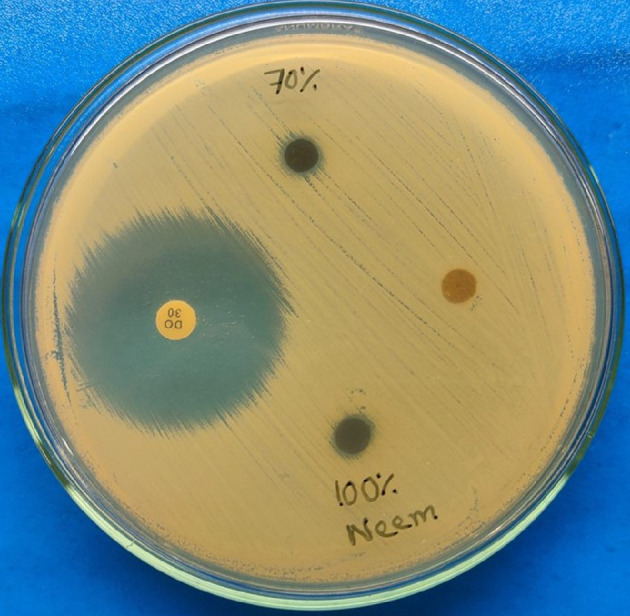
Disc-diffusion method with ethanolic extracts of selected plant parts against *S. aureus*. Here, a 6 mm disc was soaked with 100 and 70% concentrations of *A. indica* (neem), and their efficacy in inhibiting the bacteria was observed. It showed very little inhibition.

In order to determine whether plant extracts have any antibacterial properties, the disc-diffusion method was used [50]. A lawn was made on a Mueller Hinton agar (MHA) plate with a sterile cotton swab. Following a drying time of 15 min for the plates, the discs, impregnated with plant extract, were transferred onto the surface of the MHA medium, with each test plate containing four discs (figure 2). Among them, an antibiotic disc was kept as the positive control. Another one containing the respective vehicle was a negative control, and the rest of the discs contained two concentrations (100 and 70%) of neem (*A. indica*) extract [54]. In this experiment, doxycycline was used as the positive control, depending on the docking result as well as the antibiotic profile of the isolated *S. aureus* strain. Each plate also contained four treated discs that were positioned almost equally apart from the controls. For the next 24 h, the plate was kept at 37°C in the incubator and checked gently after 6, 12 and 24 h, and the zone of inhibition caused by the plant extracts was measured.

## 3. Results

### 3.1. *In silico* results

#### 3.1.1. Analysis of the homology model of the target protein, Staphopain B

**Figure 3.**
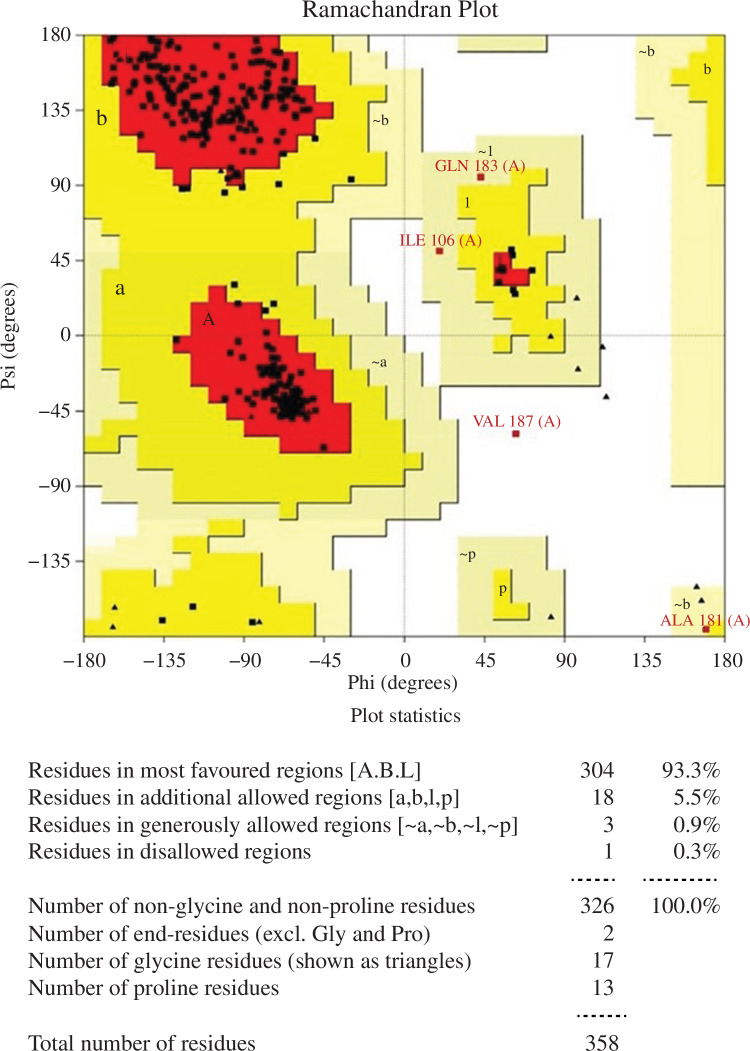
Ramachandran plot of model 3 of the Staphopain B protein from *S. aureus*. 93.3% of residues remained in favoured region; 5.5% of residues stayed in additional allowed regions; generously allowed regions contained 0.9% of residues; and 0.3% of residues remained in disallowed regions. 304 amino acids from 358 amino acid residues remained in the favoured region.

**Figure 4.**
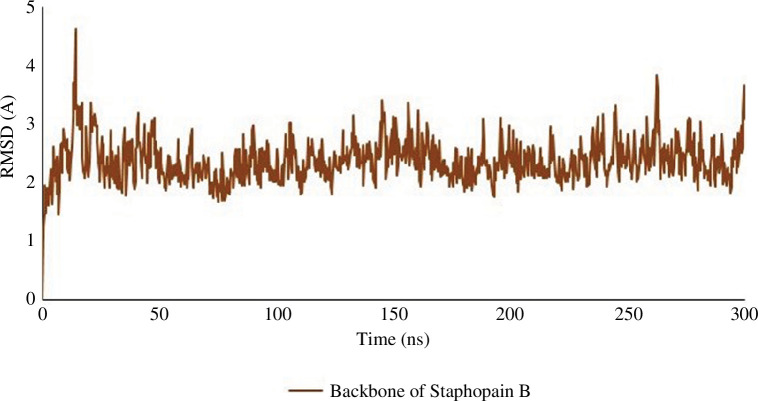
300 ns simulation graph of the RMSD of the target protein Staphopain B.

Five models (model 1, model 2, model 3, model 4 and model 5) of the protein Staphopain B were derived from the Modeller tool and the models were validated based on their structure, several angles like torsion angles, steric clashes between atoms, etc. by discrete optimized protein energy (DOPE) value, molprobity score, Ramachandran favoured and errat score [49,55,56]. From the five models, model 3 was selected for further evaluation (table 1) considering its promising features (figure 3), and the RMSD of the protein was 2.376 Å in MD simulation (figure 4).

**Table 1.** Structural characteristics of models generated from the Staphopain B amino acid sequences. Five models were derived using Modeller. Lower DOPE, molprobity score and higher values of Ramachandran favoured, and Errat were considered for the selection procedure.

model	DOPE score	molprobity	Ramachandran favoured	Errat
model 1	−39248.43	2.78	90.8%	75.65
model 2	−39228.35	3.00	90.2%	77.71
model 3	−39875.50	2.53	93.3%	81.38
model 4	−39546.59	2.92	94.2%	79.43
model 5	−39592.16	3.01	92.6%	73.43

#### 3.1.2. Twenty-five phytocompounds exhibited drug-like properties

One hundred and fifteen phytocompounds were selected from *Gingiber officinale*, *Carica papaya*, *Ocimum sanctum*, *A. indica*, *Aloe vera*, *Lawsonia enermis*, *Terminalia chebula*, *Psidium guajava*, *Senna alexandrina*, *Elaeocarpus serratus*, *Clerodendrum infortunatum*, *Adhatoda justicia*, *Phyllanthus emblica* and *Moringa oleifera* and their drug-like features were determined (electronic supplementary material, table S1). Twenty-five out of 115 phytocompounds (table 2) did not show any violation (0 violation) regarding the rules of Lipinski, Muegge, Egan, Veber and Ghose, and all of them fulfilled the parameters such as molecular weight should be below 500 g mol^−1^, and the number of hydrogen donors and acceptors should be less than or equal to 5 and 10.

**Table 2.** Pharmacological properties of the top hit phytocompounds.

phytocompounds	MW (g mol^−1^)	rotatable bonds	H-bond acceptors	H-bond donors
capsaicin	305.41	10	3	2
*n*-heptane	202.33	6	2	0
1,8-dihydroxyanthracene	210.23	0	2	2
aloe-emodin	270.24	1	5	3
1,3-dihydroxy-6,7-dimethoxyxanthone	288.25	2	6	2
anisotine	349.38	4	4	1
vasicinol	204.23	0	3	2
vasicol	206.24	2	2	2
vasicoline	291.39	2	1	0
vasicolinone	305.37	2	2	0
17-epiazadiradione	450.57	3	5	0
nimbandiol	456.53	4	7	2
6-deacetylnimbinene	440.53	4	6	1
azadiradione	450.57	3	5	0
nimbinin	466.57	3	6	0
cimbinone	286.37	0	3	1
*cis*-linalool oxide	212.29	3	3	0
thiamine	265.35	4	3	2
beta-nimolactone	386.48	2	5	0
citronellyl isobutyrate	226.36	8	2	0
niazimin A	383.39	9	8	3
apigenin	270.24	1	5	3
desfuranoazadiradione	384.51	2	4	0
nimbolide	466.52	4	7	0
epoxyazadiradione	466.57	3	6	0

#### 3.1.3. Twelve out of 25 phytocompounds passed the ADMET properties

The ADMET properties of the 25 compounds were determined (electronic supplementary material, table S2). Among them, 12 compounds did not reveal any toxicity, such as AMES toxicity and hepatotoxicity (table 3). All the ligands showed higher intestinal absorption. Moreover, almost all the ligands demonstrated water solubility and were able to permeate Caco−2 lines of cell. Regarding distribution, all compounds displayed moderate distribution in the brain. Only three compounds, nimbandiol, nimbinone and apigenin, work as substrates of P-glycoprotein. On the contrary, only four compounds, vasicinol, nimbinone, *cis*-linalool oxide and apigenin, were unable to inhibit P-glycoprotein and interestingly, nimbandiol played as both substrate and inhibitor. Most of the substances did not inhibit the members of the cytochrome P450 enzyme superfamily, including CYP1A2, CYP2C19 and CYP3A4 during metabolism and there was no compound that worked as a substrate for CYP2D6. These play pivotal roles in the metabolism of drugs [57].

**Table 3.** ADMET features of the top hit phytocompounds.

Staphopain B docking with	water solubility	Caco−2 permeability	intestinal absorption (human)	P-gp substrate	P-gp I inhibitor	blood–brain barrier permeability	CYP1A2 inhibitor	CYP2C19 inhibitor	CYP3A4 inhibitor
vasicinol	−2.54	1.14	80.40	no	no	−0.29	no	no	no
17-epiazadiradione	−5.11	0.85	98.70	no	yes	−0.19	no	no	yes
nimbandiol	−4.88	0.82	93.36	yes	yes	−0.64	no	no	yes
azadiradione	−5.11	0.85	98.70	no	yes	−0.19	no	no	yes
nimbinin	−4.3	0.91	98.65	no	yes	−0.43	no	no	yes
nimbinone	−3.23	1.31	93.59	yes	no	−0.20	no	yes	no
*cis*-linalool oxide	−1.97	1.64	97.29	no	no	0.15	no	no	no
apigenin	−3.33	1.01	93.25	yes	no	−0.73	yes	yes	no
desfuranoazadiradione	−4.73	1.35	98.36	no	es	−0.36	no	no	no
epoxyazadiradione	−4.3	0.91	98.65	no	yes	−0.43	no	no	yes
nimbolide	−5.17	0.92	100.00	no	yes	−0.68	no	no	no
beta-nimolactone	−4.62	1.11	97.42	no	yes	−0.23	no	no	no

#### 3.1.4. Interpretation of molecular docking (by Autodock-Vina) outcomes

The molecular docking outcomes of the antibiotics with Staphopain B are described in table 4. The antibiotic (doxycycline) with the highest binding affinity score of −7 kcal mol^−1^ was chosen as the control, and among 12 molecules (electronic supplementary material, table S3), 9 molecules showed higher binding affinity than the control drug (table 5).

**Table 4.** The binding affinity between Staphopain B and commercially available drugs of *S. aureus*.

docking with	read-1 (kcal mol^−1^)	read-2 (kcal mol^−1^)	read-3 (kcal mol^−1^)	average (kcal mol^−1^)
doxycycline	−6.9	−6.8	−7.3	-7
cefazolin	−6.7	−6.5	−5.8	−6.33
nafcillin	−6.6	−6.8	−6.8	−6.73
linezolid	−6.4	−6.2	−6.2	−6.27
trimethoprim	−5.9	−5.2	−5.5	−5.53
clindamycin	−5.9	−6.3	−6.4	−6.2
oxacillin	−7.4	−7.3	−6.5	−6.93
aztreonam	−5.3	−6.1	−5.9	−5.77

**Table 5.** The binding affinity between Staphopain B and the most promising phytocompounds.

docking with	read-1 (kcal mol^−1^)	read-2 (kcal mol^−1^)	read-3 (kcal mol^−1^)	average (kcal mol^−1^)
nimbolide	−8.3	−8.2	−8.2	−8.23
nimbinin	−7.7	−7.7	−7.7	−7.70
epoxyazadiradione	−7.7	−7.7	−7.7	−7.70
azadiradione	−7.6	−7.8	−7.5	−7.63
beta-nimolactone	−7.5	−7.5	−7.5	−7.50
nimbinone	−7.3	−7.4	−7.4	−7.37
apigenin	−7.3	−7.3	−7.3	−7.30
desfuranoazadiradione	−7.3	−7.4	−7.1	−7.27
17-epiazadiradione	−7.2	−7.2	−7.1	−7.17

#### 3.1.5. Interpretation of molecular docking (by Swissdock server) outcomes

**Figure 5.**
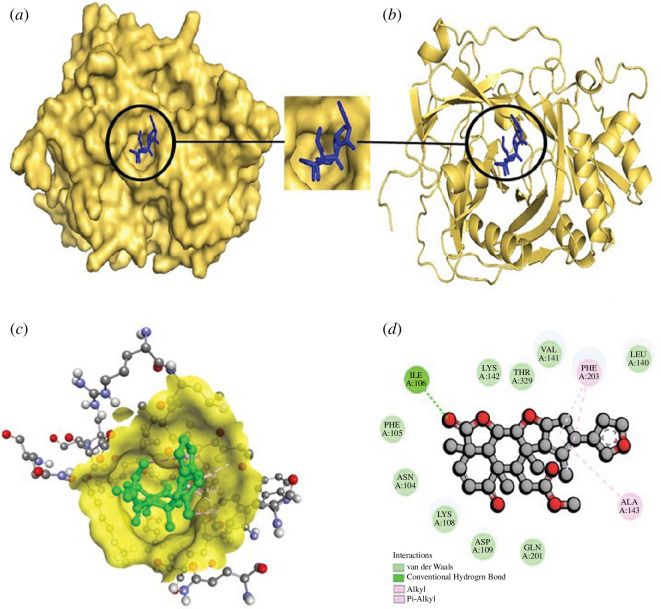
The structural depiction of the Staphopain B_Nimbolide complex. (*a*) Surface view of the Staphopain B_Nimbolide complex. Here, the yellow colour indicates protein and the blue colour demonstrates ligand. (*b*) Pose view of the Staphopain B_Nimbolide complex. (*c,d*) Three- and two-dimensional interactions of the Staphopain B_Nimbolide complex. Here, protein and ligand are expressed in yellow and green colour, respectively.

**Figure 6.**
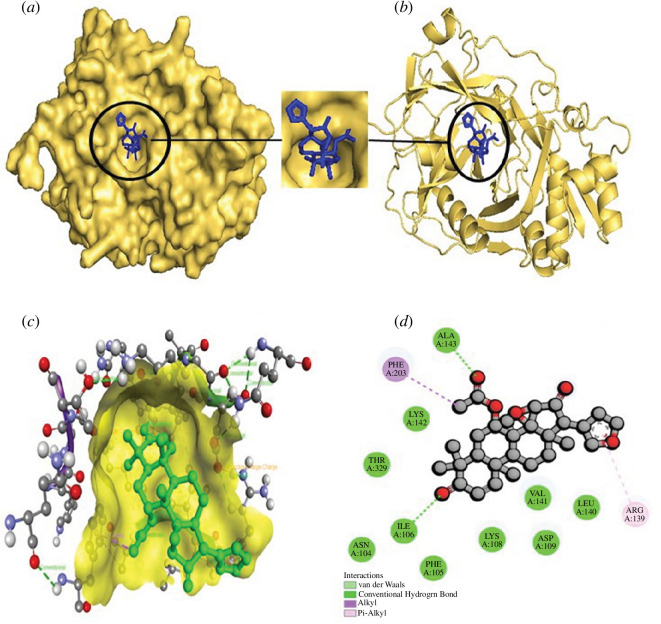
The structural depiction of the Staphopain B_Epoxyazadiradione complex. (*a*) Surface view of the Staphopain B_Epoxyazadiradione complex. Here, the yellow colour indicates protein and the blue colour demonstrates ligand. (*b*) Pose view of the Staphopain B_ Epoxyazadiradione complex. (*c,d*) Three- and two-dimensional interactions of the Staphopain B_ Epoxyazadiradione complex. Here, protein and ligand are expressed in yellow and green colour, respectively.

**Figure 7.**
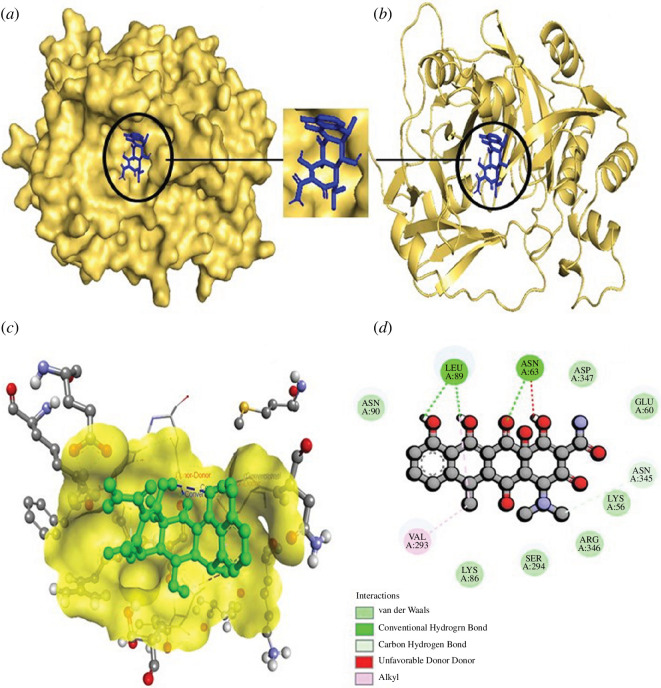
The structural depiction of the Staphopain B_Doxycycline complex. (*a*) Surface view of the Staphopain B_ Doxycycline complex. Here, the yellow colour indicates protein and the blue colour demonstrates ligand. (*b*) Pose view of the Staphopain B_ Doxycycline complex. (*c,d*) Three- and two-dimensional interactions of the Staphopain B_ Doxycycline complex. Here, protein and ligand are expressed in yellow and green colour, respectively.

The fitness and the estimated scores of ΔG of nine phytocompounds along with antibiotics were determined. Among them, the estimated ΔG score of nimbolide (figure 5) and epoxyazadiradione (figure 6) was lower than −7.8 kcal mol^−1^, which was the estimated ΔG score of doxycycline (figure 7). Moreover, the full fitness and hydrogen bond interactions of these compounds with various amino acids were also evaluated as potential drug candidates (table 6).

**Table 6.** Estimated ΔG, full fitness, nd hydrogen bonds of the selected drug-like molecules and their interaction with Staphopain B.

docking with	estimated Δ*G* (kcal mol^−1^)	full fitness (kcal mol^−1^)	hydrogen bonds	interacting amino acid
nimbolide	−7.93	−1754.01	3	ILE106, PHE105, VAL141
nimbinin	−7.16	−1536.19	2	ILE106, ALA143
epoxyazadiradione	−7.91	−1542.81	2	ILE106, ALA143
azadiradione	−7.33	−1777.70	2	ILE106, ALA144
beta-nimolactone	−7.28	−1797.98	2	ASP109, ILE106
nimbinone	−7.09	−1815.47	2	ALA51, LYS21
apigenin	−7.17	−1840.82	3	ASN104, ASP328
desfuranoazadiradione	−7.43	−1789.43	2	ILE106, ALA143
17-epiazadiradione	−7.60	−1776.57	2	ILE106, ALA143
doxycycline	−7.8	−1793.73	3	ASP109, LEU140, ASP109

#### 3.1.6. Analysis of the outcome of the molecular dynamics simulation

**Figure 8.**
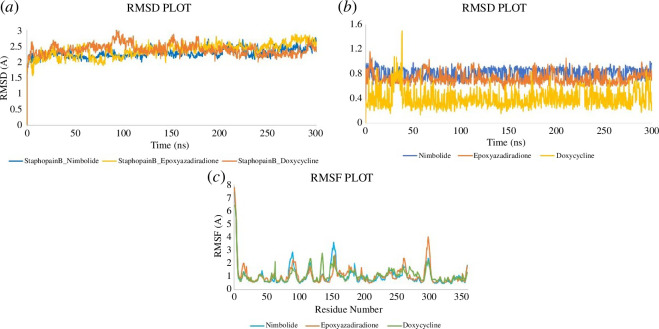
Illustration of simulation curves. The graphs show the characteristics of (*a*) RMSD of the backbone of the protein regarding ligands, (*b*) RMSD of ligands, and (*c*) RMSF at 300 ns of MD simulation with the help of Desmond software.

Root mean square deviation (RMSD), root mean square fluctuation (RMSF), ligand behaviour and protein–ligand interaction were retrieved from the MD simulation. The result interpretation of RMSD expresses the stability of the protein–ligand complexes. The average RMSD plots of the backbone of Staphopain B regarding nimbolide, epoxyazadiradione and doxycycline were 2.282 Å, 2.376 Å and 2.394 Å (figure 8*a*). Throughout the entire 300 ns simulation period, the change of the curve remained below 3.00 Å, indicating that the protein–ligand interaction was stable [44]. Average RMSD values of 0.818, 0.73 and 0.408 Å for the ligands nimbolide, epoxyazadiradione and doxycycline, respectively, showed a steady conformation with protein (figure 8*b*). Furthermore, the average RMSD value of the ligands was good compared to the control. Likewise, the average RMSF values of Staphopain B_Nimbolide, Staphopain B_Epoxyazadiradione and Staphopain B_Doxycycline were 1.034, 1.097 and 1.08 Å, respectively. Besides, the N- and C-terminal zones, a greater volatility of around 4 Å was seen between 80 and 310 residues (figure 8*c*).

**Figure 9.**
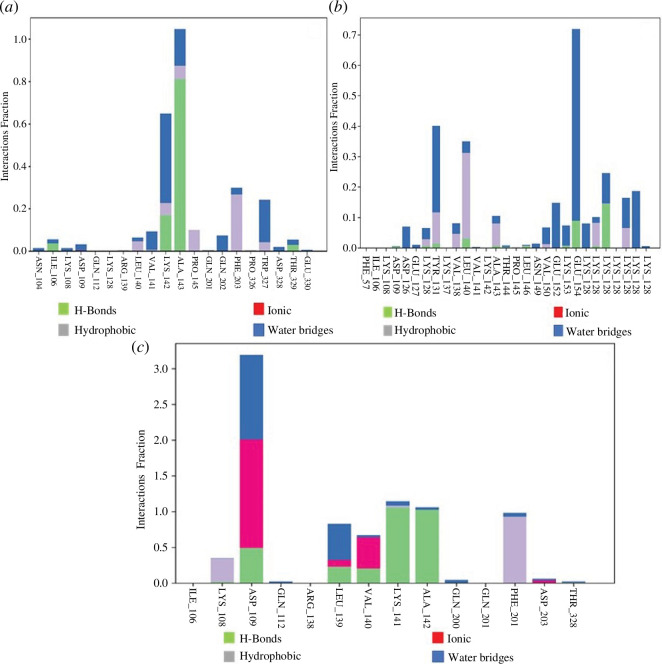
Histogram of Staphopain B–ligand complex chart of (*a*) Staphopain B_Nimbolide, (*b*) Staphopain B_Epoxyazadiradione and (*c*) Staphopain B_Doxycycline. The interaction fraction of (*a,b*) is less than 1, but in (*c*), it is greater than 1 owing to more than one interaction of protein with ligand.

Non-covalent interactions between proteins and ligands were measured during a period of 300 ns. Nimbolide produced LYS142 (hydrogen bonds, hydrophobic and water bridges), ALA143 (hydrogen bonds, hydrophobic and water bridges) and PHE203 (hydrophobic and water bridges) interactions with Staphopain B for 65%, 105% and 30% of the 300 ns time frame (figure 9*a*). Epoxyazadiradione ligand made bonds with TYR131 (hydrogen bonds, hydrophobic and water bridges), LEU140 (hydrogen bonds, hydrophobic and water bridges) and GLU154 (hydrogen bonds and water bridges) for 40%, 38% and 70%, respectively, of the simulation (figure 9*b*). Furthermore, doxycycline interacted with ASP109 (hydrogen bonds, ionic bonds and water bridges), LEU139 (hydrogen bonds, ionic bonds and water bridges), VAL140 (hydrogen bonds, ionic bonds and water bridges), LYS141 (hydrogen bonds and water bridges), ALA142 (hydrogen bonds and water bridges) and PHE202 (hydrophobic bonds and water bridges) for 325%, 70%, 75%, 110%, 100% and 100% of simulation (figure 9*c*). It is not impossible for interaction fraction values to exceed 1.0, given that certain protein residues may establish more than one contact of the same type with the ligand [58].

### 3.2. *In vitro* results

#### 3.2.1. *Staphylococcus aureus* strain was isolated and identified *in vitro*

**Figure 10.**
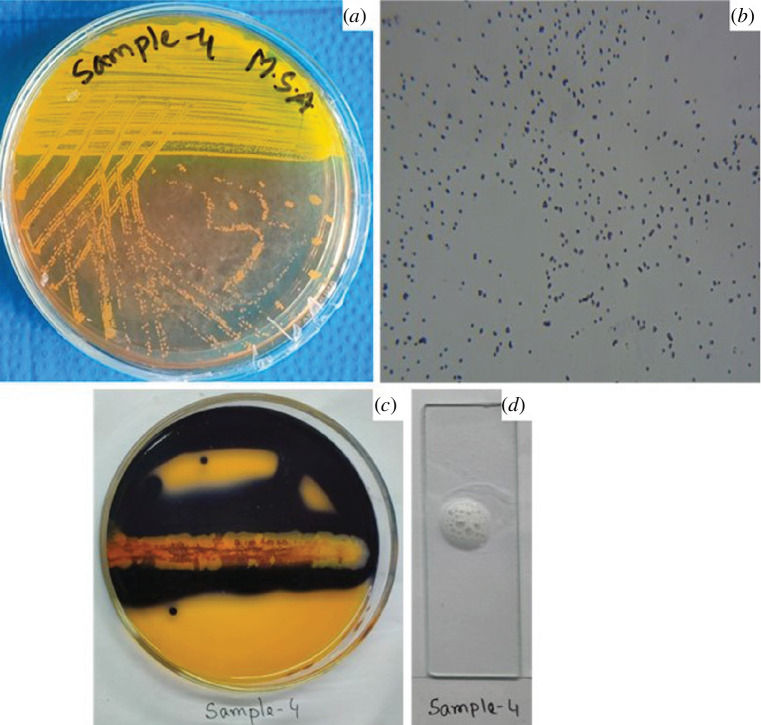
Representation of several biochemical tests of the *S. aureus* strain, which was designated as sample-4. (*a*) The strain fermented mannitol in MSA media. (*b*) The strain showed purple colour in the Gram staining procedure. (*c,d*) Positive results in starch hydrolysis and catalase tests, respectively.

*Staphylococcus aureus* was isolated from the pus samples collected from the discharge of skin infections in patients. The strain was verified through positive result in Gram staining as well as positive result in catalase and hydrolase test and it has also fermented mannitol in MSA media, which is identical media for *S. aureus* (figure 10).

#### 3.2.2. The isolated strain showed resistance to aztreonam and oxacillin but sensitivity to doxycycline

**Figure 11.**
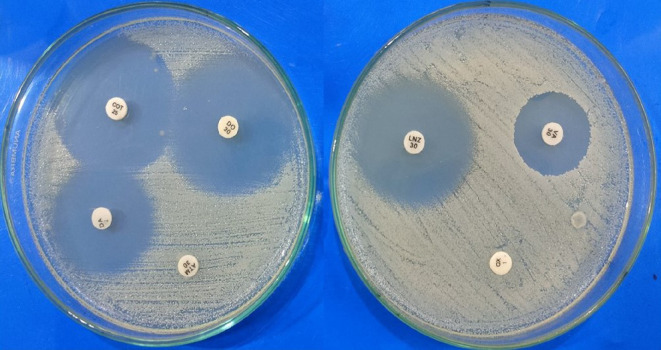
Sensitivity patterns of *S. aureus* to common antibiotics.

Like *in silico* assessment, doxycycline also showed better inhibition of *S. aureus* than other antibiotics (figure 11). Among the seven conventional antibiotics used frequently in hospital settings, the isolated *S. aureus* showed a differential sensitivity pattern (table 7). According to CLSI guidelines, the organism displayed the highest sensitivity to doxycycline (30 mm), followed by co-trimoxazole (29 mm), linezolid (27 mm) and vancomycin (20 mm). On the other hand, it has represented absolute resistance to aztreonam and oxacillin. According to the sensitivity pattern, the organism exhibited moderate sensitivity to clindamycin.

**Table 7.** Representation of the susceptibility of *S. aureus* to seven commercial antibiotics. Millimetres served as the unit of measurement for the antibiotics’ zone of inhibition. The reaction of the organism to the antibiotics was either sensitive, intermediate or resistant. The table demonstrates that while the organism was sensitive to doxycycline and linezolid, it had established the strongest resistance to aztreonam and oxacillin.

antibiotic	inhibition zone	comments	zone diameter interpretive standard (mm)		
			sensitive	intermediate	resistant
doxycycline 30 (DO)	30	sensitive	≥16	13–15	≤12
aztreonam 30 (ATM)	0	resistant	≥21	18–20	≤17
linezolid 30 (LNZ)	27	sensitive	≥21	20.1–20.9	≤20
clindamycin 2 (DA)	18	intermediate	≥21	15–20	≤14
vancomycin 30 (VA)	20	sensitive	≥17	15–16	≤14
oxacillin 1 (OX)	0	resistant	≥13	11–12	≤10
co-trimoxazole 25 (COT)	29	sensitive	≥16	11–15	≤10

#### 3.2.3. Analysis through spectrophotometer

**Figure 12.**
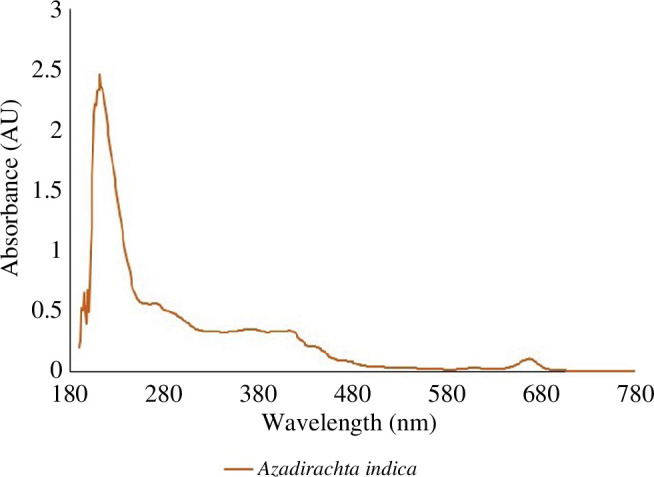
UV absorption spectrum of the crude phytoextract revealing *A. indica* characteristics. Absorption was detected across wavelengths from approximately 190 to 460 nm, indicating the presence of diverse compounds including alkaloids, terpenoids and phenolic compounds. A prominent peak absorbance (around 2.5) was observed at 200–210 nm.

Owing to the sharpness of the peaks and the appropriate baseline, the UV–visible profile of the plant extracts was collected in the wavelength range of 190–1100 nm. Compounds with specific chemical bonds, chromophores, lone pairs of electrons and aromatic rings were found by analysing their UV–visible spectra. All the extracts showed peaks at 190–703 nm, which revealed the presence of phenolic compounds, terpenoids and glycoside compounds (figure 12) [59].

#### 3.2.4. High-performance liquid chromatography analysis of the extract

**Figure 13.**
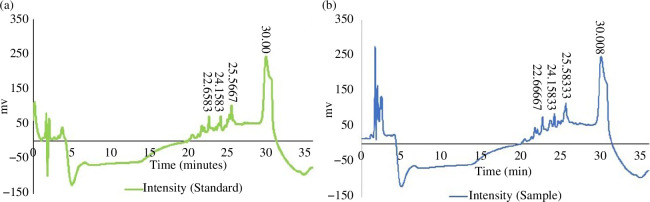
HPLC chromatograms of the leaf extract of *A. indica*. The chromatogram of the separated standard at several retention times at 217 nm wavelength (*a*) indicated similarity with the peaks given by the plant extract at different retention times at the same wavelength (*b*).

HPLC analysis was done in order to identify the constituents remaining in the leaf extract. The chromatograms of the HPLC are demonstrated in figure 13. The outcome manifested that the standard (std) nimbolide showed several eaks at various retention times (22.66, 24.16, 25.57 and 30.00 min) (figure 13*a*). On the other hand, the compound was present in the extract, which had retention periods of 22.67, 24.16, 25.58 and 30.01 min, respectively (figure 13*b*).

#### 3.2.5. Analysis of the antibacterial sensitivity pattern of phytocompounds

Though 100 and 70% concentrations of the ethanolic extract of leaves of *A. indica* showed some inhibition against *S. aureus*, it is lower than that of the control antibiotic (figure 2).

## 4. Discussion

*Staphylococcus aureus* has been designated as a priority pathogen by the World Health Organization (WHO). The organism was listed as one of the most notorious pathogens that are rapidly developing resistance against the available antibiotics [60]. In 2017, WHO expressed a list of priority pathogens called ESKAPE (*Enterococcus faecium*, *S. aureus*, *Klebsiella pneumoniae*, *Acinetobacter baumannii*, *Pseudomonas aeruginosa* and *Enterobacter* species), and declared the urgency of developing new antibiotics against these organisms, thereby emphasizing direct research linked to drug development [61]. *Staphylococcus aureus* can cause a wide variety of diseases and it is becoming increasingly resistant to multiple antibiotics. In order to inform public health policies and actions, WHO also highlights the significance of surveillance and monitoring of *S. aureus* infections and resistance patterns [10]. Furthermore, to combat the growing threat posed by multi-drug-resistant *S. aureus*, the organization has urged the development of new medicines and other therapies, as well as enhanced infection prevention and control procedures, to lessen the spread of the bacterium in healthcare and community settings [62]. For this purpose, *in silico* or computational-based drug design may be a blessing because it is an essential tool in modern drug discovery, allowing researchers to rapidly screen and optimize potential drug candidates, saving time and resources in the drug development process, and ultimately helping to bring safe and effective drugs to market more quickly [63]. Therefore, the current research to find new drug-like compounds against *S. aureus* via *in silico* screening followed by *in vitro* evaluation is important and timely. Drugs can be made from chemically synthesized molecules and natural compounds derived from plants. Compared with their manufactured chemical counterparts, these phytochemicals found in plants are nearly harmless [64]. Despite the potential benefits of phytocompound-based drugs, their production and use in Bangladesh are still limited [65]. *Azadirachta indica*, a traditional medicinal plant of Bangladesh, has considerable antibacterial action against *S. aureus* and other pathogens. Many phytocompounds, including nimbolide, azadirachtin, epoxyazadiradione, nimbinin, etc., were isolated from the neem extract and shown to be responsible for its antibacterial activity in previous studies [66]. Therefore, the appearance of neem-based phytocompounds from the *in silico* part of the study was not surprising. Moreover, some other traditional plants, such as *Carica papaya*, *Justicia adhatoda*, *Clerodendrum infortunatum*, etc., have also contributed to treating several diseases, such as malaria, analgesics, jaundice, etc. [67,68]. Especially, papaya and basak are frequently used for numerous ayurvedic treatments [69]. In the present research, the 115 phytocompounds from the 14 medicinal plants were screened from the database and tested for their pharmacological and ADMET properties. Following that, their efficiency against the predetermined important proteins from *S. aureus* was evaluated.

Targeting the potential protein is essential for the drug development process. If a protein’s action can be bypassed, targeting that one would be less likely to end up with a success story. Staphopain B was selected based on its functions, virulence factors and stability in MD simulation (figure 4). Hence, since the protein displayed lower RMSD (2.376 Å) at the initial MD simulation, it is likely that the protein will remain stable during the simulation period with ligand; interestingly, the later simulation data (2.282, 2.376 and 2.394 Å for nimbolide, epoxyazadiradione and doxycycline, respectively, with the protein) supported this assumption. Since the target protein, Staphopain B, has an identical template (1X9Y), homology modelling was done with the help of Modeller. Available templates increase the chance of building a model and finding reference models [70]. Then, the model was refined and validated to repair the missing residues and make the protein more stable. After detecting the active sites of the protein (sequence nos. 54, 66, 80, 101, 102, 103, 105, 130, 142,144, 323, 325, 327, 328, 332, 341, 342, 343 and 344), docking was performed with 12 selected phytocompounds. These phytocompounds did not show any violation of the drug-likeness rules of Lipinski, Muegge, Egan, Veber and Ghose and did not demonstrate any toxicity, such as AMES toxicity and hepatotoxicity, at the time of analysing ADMET properties. Docking confirms the appropriate attachment of a ligand to the binding pocket of a protein molecule [71]. In the study, the control drug, doxycycline, was not determined only from references. Rather, eight antibiotics, which were suggested in much previous research and are often used to treat *S. aureus* infections, were docked with the determined target protein to select a control drug. Among the 12 phytocompounds, 9 compounds revealed more affinity than the control drug (−7 kcal mol^−1^), thereby indicating their potentiality in drug development. Eventually, two compounds among them exhibited a higher ΔG score (nimbolide, −7.93 kcal mol^−1^; epoxyazadiradione, −7.91 kcal mol^−1^) than the control (−7.8 kcal mol^−1^), and the number of hydrogen bonds (nimbolide, 3; epoxyazadiradione, 2; doxycycline, 3) was also studied. These properties indicate whether the selected compounds are safe, stable and can serve the desired purpose, i.e. have potential in drug development. In comparison to the control compound, doxycycline, nimbolide and epoxyazadiradione were selected for MD simulation, and they showed almost the same result and, in some aspects, better results.

To evaluate the efficacy of the selected phytocompound, *in vitro* evaluation was performed with the relevant plant extracts. To try all the stones, extracts were prepared from *A. indica* leaves linked to the phytocompounds that showed higher affinity than doxycycline. Ethanolic extracts were prepared by the Soxhlet apparatus for the assessment, with the aim of bringing compounds into solution that are insoluble in water. Hence, the final phytocompounds nimbolide and epoxyazadiradione are tetranortriterpenoid and limonoid, respectively [72,73], and ethanol was the solvent of choice for the assay [53,74]. The solvent was evaporated through the rotary evaporator, which was then employed at 70 and 100% concentrations in bacterial lawn. The bacterial strain was isolated from a human pus sample and grown on selective media, specifically MSA [75]. It appeared Gram-positive and exhibited mannitol fermentation, indicative of *S. aureus* characteristics. Additionally, it hydrolysed starch and tested positive in the catalase test [75,76]. The preparation of an antibiogram displays the antibiotic profile of the isolated bacteria, which strengthens the merit of the study. Empiric antimicrobial treatment is frequently guided by antibiograms, which are also used to detect and track patterns of antimicrobial resistance. It offers details on how bacteria react to various antibiotics, assisting medical professionals in selecting the best antibiotic for the infection [77]. Notably, the *in silico* evaluation and *in vitro* assessment both agreed in determining doxycycline as the central drug for *S. aureus* in the present study (tables 4 and 7). The use of appropriate controls (doxycycline as a positive control and a disc with an equivalent amount of ethanol as a negative control) represents the standards for the assay. Although previous studies evidenced the effectiveness of the subjected plant extracts against *S. aureus* [66], this research, however, did not observe the expected area of inhibition—a narrow inhibition zone (8.1 mm, 7.5 mm) occurred at both 100% and 70% neem extract (figure 2).

The UV visualization of the leaf extract showed the presence of the phytochemicals, and the HPLC outcomes support their presence in the plant extract. The peaks at 217 nm at 30 min time are those of the desired compound, nimbolide, which was found in the present study too [53]. The plant extract showed effective inhibition in the *in silico* method (tables 5 and 6), but the plant extracts revealed little inhibition in the *in vitro* method. This unexpected outcome may have several explanations, such as that the phytocompounds may remain in a bound state and therefore fail to interact in the reaction, the higher concentration may leave different improved effects, or other biological solvents could have positive effects. In this research, the ethanolic extract of *A. indica* (neem) started to show inhibition, but it could not continue because of the lower amount of desired phytocompounds in the extract. This could be the sole reason for lower inhibition. Therefore, further evaluation of the phytocompounds performed using formulated compounds could eliminate the doubt of this present inhibition assay.

The present research outcomes suggest nimbolide and epoxyazadiradione as potential phytocompounds that could be exploited for new drug development to treat *S. aureus*, given that necessary *in vitro* and *in vivo* evaluations are performed.

## 5. Conclusion

*In silico* screening, in tandem with molecular docking studies, has proven to be a successful and accurate method for identifying potential drug candidates; molecular dynamics simulation has added the benefit of refining and optimizing the target protein and studying the binding affinity of the protein–phytocompound complex. Here, our identified phytocompounds exhibited favourable drug-likeness properties, including low toxicity and high bioavailability. Moreover, *S. aureus* was isolated from clinical samples and identified via physical, biochemical and molecular characterization, its relationship with close species was determined, and its antibiotic profile was assessed *in vitro*. Furthermore, the efficacy of the screened phytocompounds to inhibit the growth of the bacteria was evaluated. Despite the limited inhibitory activity of the plant extracts, the findings, therefore, highlight the potential of natural compounds as sources for the development of novel drugs against the pathogen. Finally, this study provides a foundation for future investigations into the development of natural compound-based therapeutics for the treatment of *S. aureus* infections. However, further experimental research is required to confirm the phytocompounds’ effectiveness *in vivo* and to evaluate their potential for clinical application.

## Data Availability

New data are presented in the study; supplementary data obtained from this study are provided both in the main paper and in supplementary tables:https://figshare.com/articles/dataset/Supplementary_tables_docx/24243409. Supplementary material is available online [78].
